# Branched-Chain and Aromatic Amino Acids Mark Early Metabolic Shifts in Adults with Varying Adiposity

**DOI:** 10.3390/ijms27135912

**Published:** 2026-06-30

**Authors:** Marta Jaskulak, Iwona Rybakowska, Magdalena Gregorczyk, Klaudia Antoniak-Pietrynczak, Anna Sośnicka, Patrycja Jabłońska, Katarzyna Zorena

**Affiliations:** 1 Department of Immunobiology and Environment Microbiology, Faculty of Health Sciences, Institute of Maritime and Tropical Medicine, Medical UnIiversity of Gdansk, Dębinki 7, 80-211 Gdansk, Poland; 2 Department of Biochemistry and Clinical Physiology, Medical University of Gdansk, Debinki 1, 80-211 Gdansk, Polandmagdalena.gregorczyk@gumed.edu.pl (M.G.); 3 Department of Biochemistry, Medical University of Gdansk, Debinki 1, 80-211 Gdansk, Poland

**Keywords:** obesity, insulin resistance, HOMA-IR, amino acids, liquid chromatography-mass spectrometry

## Abstract

Amino acid metabolism has been increasingly recognized as a central determinant of obesity and insulin resistance, yet the specific contributions of individual amino acids require further clarification. The aim of the study was to detect relationships between serum amino acid concentrations and metabolic parameters in overweight and obese individuals. Amino acid concentrations were measured in 50 individuals classified as normal weight, overweight, or obese, and were analyzed using principal component analysis (PCA), K-means clustering, multiple linear regression, and Random Forest models. Obese individuals exhibited markedly elevated levels of branched-chain amino acids (BCAAs: valine, isoleucine, leucine) and glutamic acid, accompanied by reduced concentrations of serine, glycine, and glutamine, compared with normal weight participants. PCA revealed that the first component, which explained 35.5% of the total variance, was driven primarily by BCAAs, serine, and glutamine, while the second component, accounting for 9.5% of variance, was influenced by threonine, tryptophan, and asparagine. The multiple linear regression model explained 89.6% of the variance in HOMA-IR (R^2^ = 0.896, *p* < 0.001), with isoleucine emerging as the strongest positive predictor (*p* < 0.001), valine and leucine showing additional significant associations (*p* = 0.035), and tyrosine demonstrating a significant negative association (*p* = 0.039), while proline was not significant. The Random Forest model predicting insulin resistance achieved robust cross-validated performance (R^2^ = 0.86 ± 0.06), with valine, isoleucine, and leucine accounting for the majority of predictive importance, followed by tyrosine and glutamine. Together, these findings demonstrate that amino acid profiling provides powerful discriminatory and predictive capacity for insulin resistance and obesity. BCAAs consistently emerged as the most important predictors across complementary analytical frameworks, confirming their central role in metabolic dysregulation, while glycine appeared to exert a potential protective effect. The identification of a metabolically overweight subgroup underscores the heterogeneity of the overweight state and highlights the utility of amino acid profiling for early risk stratification and the development of targeted interventions.

## 1. Introduction

The global rise in obesity and its associated metabolic comorbidities—including insulin resistance (IR), dyslipidemia, hypertension, and type 2 diabetes mellitus (T2DM)—has become a pressing public health concern, with significant implications for morbidity, mortality, and healthcare systems worldwide [1]. According to the World Health Organization (WHO), obesity is defined as “abnormal or excessive fat accumulation that presents a risk to health” [2]. It is also known that obesity represents a complex interplay of genetic, environmental, and biochemical factors, leading to systemic metabolic dysfunction characterized by impaired insulin action, chronic low-grade inflammation, and vascular dysregulation [3,4].

Recent advances in metabolomics, particularly high-throughput platforms capable of profiling small-molecule metabolites, have provided critical insights into early biochemical perturbations that precede overt metabolic disease [5]. Among these, circulating amino acids—especially the branched-chain amino acids (BCAAs: leucine, isoleucine, and valine)—are starting to emerge as prominent biomarkers of early metabolic derangement [6]. Particularly, in recent years, high-throughput metabolomic profiling has revealed that perturbations in circulating amino acid pools—most notably BCAAs—are not merely consequences of excess adiposity but may actively contribute to the early pathogenesis of metabolic dysfunction [7]. Elevated BCAA levels have been consistently observed in obese cohorts and correlate strongly with surrogate markers of insulin resistance, including HOMA-IR and fasting insulin [8]. Mechanistic studies have further illuminated the pathophysiological role of BCAAs in metabolic dysfunction [9]. Impaired BCAA catabolism—largely due to downregulated activity of the branched-chain α-keto acid dehydrogenase (BCKDH) complex in liver, skeletal muscle, and adipose tissue—leads to mitochondrial overload and accumulation of catabolic intermediates such as C3/C5 acylcarnitine and 3-hydroxyisobutyrate [7]. These metabolites promote mTORC1 activation, serine phosphorylation of IRS-1, and impaired glucose uptake, collectively contributing to insulin resistance [10]. Moreover, BCAA-derived signaling appears to stimulate ectopic lipid deposition, further exacerbating metabolic stress [6]. Conversely, reductions in glycine and glutamine have been implicated in diminished metabolic flexibility and heightened inflammatory markers [11]. In this context, amino acids not only serve as substrates for protein synthesis but also act as signaling molecules regulating glucose and lipid metabolism [12]. Beyond BCAAs, aromatic amino acids (AAAs: phenylalanine, tyrosine, and tryptophan) and acidic metabolites (e.g., α-hydroxybutyrate, 2-aminoadipic acid) have been implicated in the subclinical progression of insulin resistance and β-cell dysfunction [13]. Elevated AAAs correlate with inflammatory tone and β-cell stress, while glycine and glutamine, often found at reduced levels in obesity and IR, have been linked to enhanced metabolic flexibility, antioxidant capacity, and insulin sensitivity. Glutamate, in particular, has emerged as a strong metabolic correlate of visceral adiposity [14].

Despite emerging consensus, most studies sample heterogeneous populations—often older or comorbid—limiting our understanding of amino acid alterations as potential early biomarkers [11]. This complicates efforts to identify early biochemical shifts that occur during the transition from a metabolically healthy state to overt insulin resistance and hypertension. The absence of comorbidities presents a unique opportunity to characterize incipient metabolic inflections before irreversible pathophysiological changes occur. The transition from normal weight to obesity in such cohorts may reveal early metabolic inflection points more subtle than diagnostic thresholds [14].

The study aims to elucidate the relationships between amino acid concentrations and key metabolic parameters—including body mass index (BMI), HOMA-IR, and fasting insulin in patients with overweight and obesity but without any comorbidities. Specifically, we employ multiple linear regression and principal component analysis (PCA) to identify amino acid predictors of insulin resistance, and to characterize metabolic phenotypes across a cohort of 50 individuals spanning normal weight to obesity.

## 2. Results

### 2.1. Clinical Characteristics of Patients with Normal Body Mass Index (Group I), Overweight Patients (Group II), and Obese Patients (Group III)

Ultimately, 50 subjects were included in the study, and the age of the study participants was 38 ± 11 years. Patients with normal body mass index were group I (n = 20), overweight patients as group II (n = 20) and obese patients as group III (n = 10). The age of the study participants was 38 ± 11 years on average in group I, 37 ± 12 years in group II, and 39 ± 11 years in group III. Group I patients had a statistically significantly lower BMI compared to the BMI of group II patients (*p* = 0.016) and compared to the BMI values of group III patients (*p* = 0.014). Also, a statistically significantly lower visceral adipose tissue (VAT) value was detected in group I patients compared to the VAT value of group II patients (*p* = 0.0015) and compared to the VAT value of group III patients (*p* = 0.001). Moreover, group I was characterized by a statistically significantly lower level of the WHR index compared to group II (*p* = 0.00001) and compared to group III (*p* = 0.0000003). In addition, significantly lower systolic blood pressure was detected in group I compared to systolic blood pressure in group III (*p* = 0.000000007). No statistically significant differences in the level of systolic blood pressure were detected between group I and group II. Group II patients had a statistically significantly lower VAT value compared to group III patients (*p* = 0.008). Group II was characterized by a statistically significantly lower WHR level compared to group III (*p* = 0.0000002). A statistically significantly lower level of systolic blood pressure was also detected in group II compared to group III (*p* = 0.0006). The clinical characteristics of the subjects are presented in Table 1.

### 2.2. The Relationship Between BMI and Amino Acid Profiles

**Figure 1:**
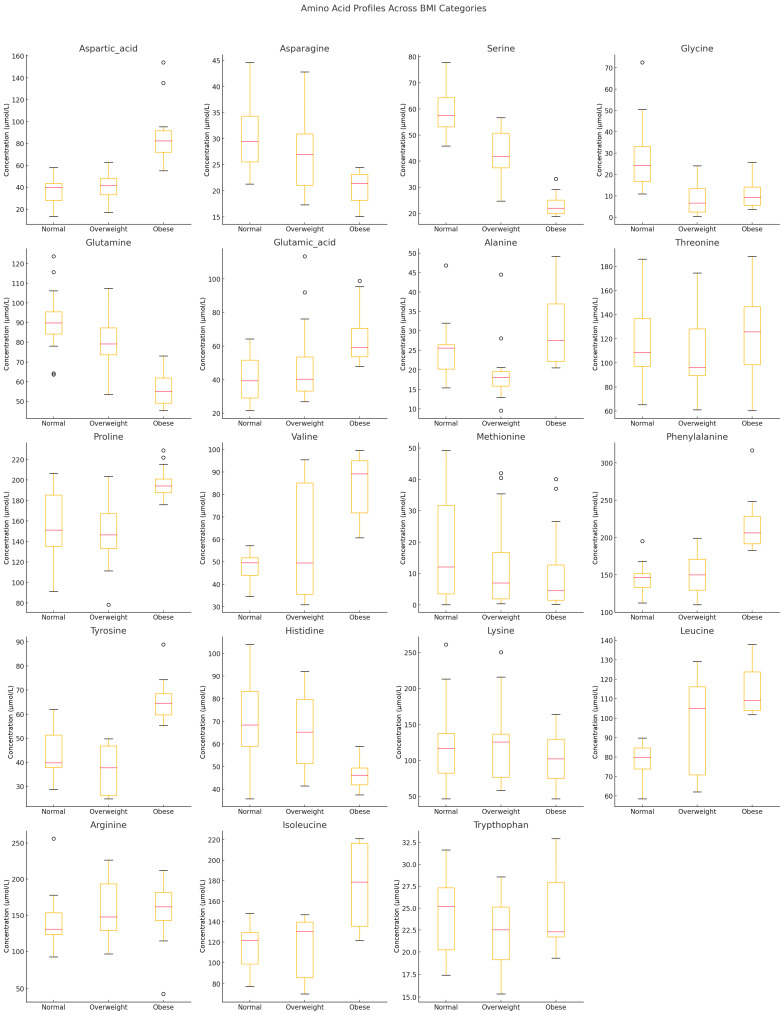

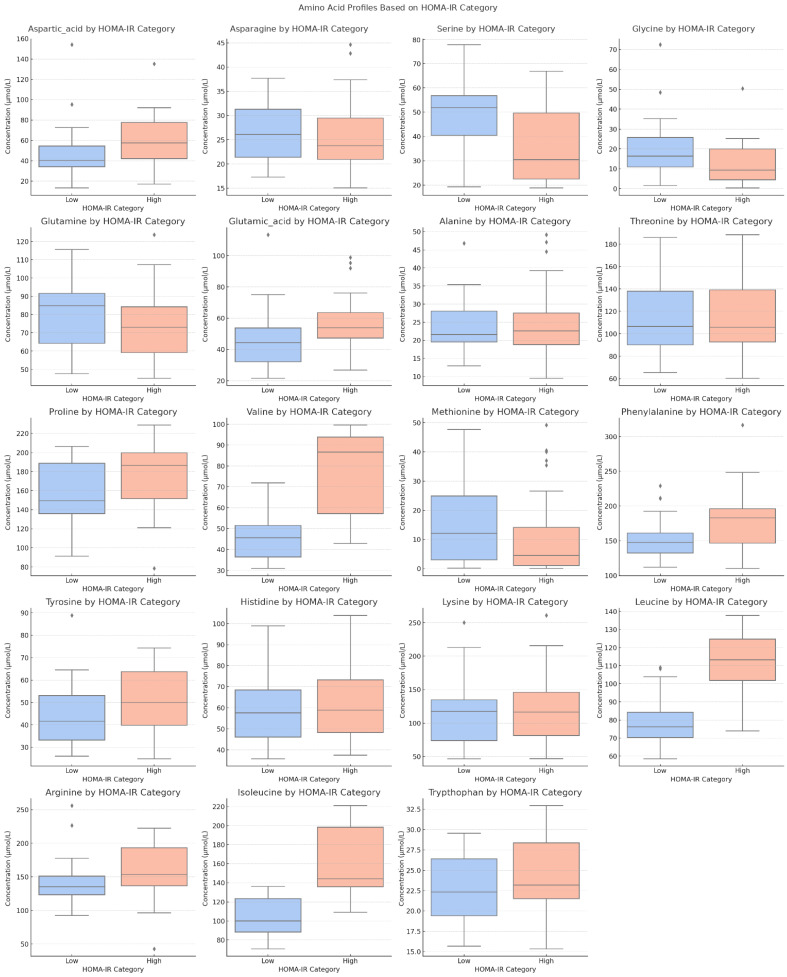

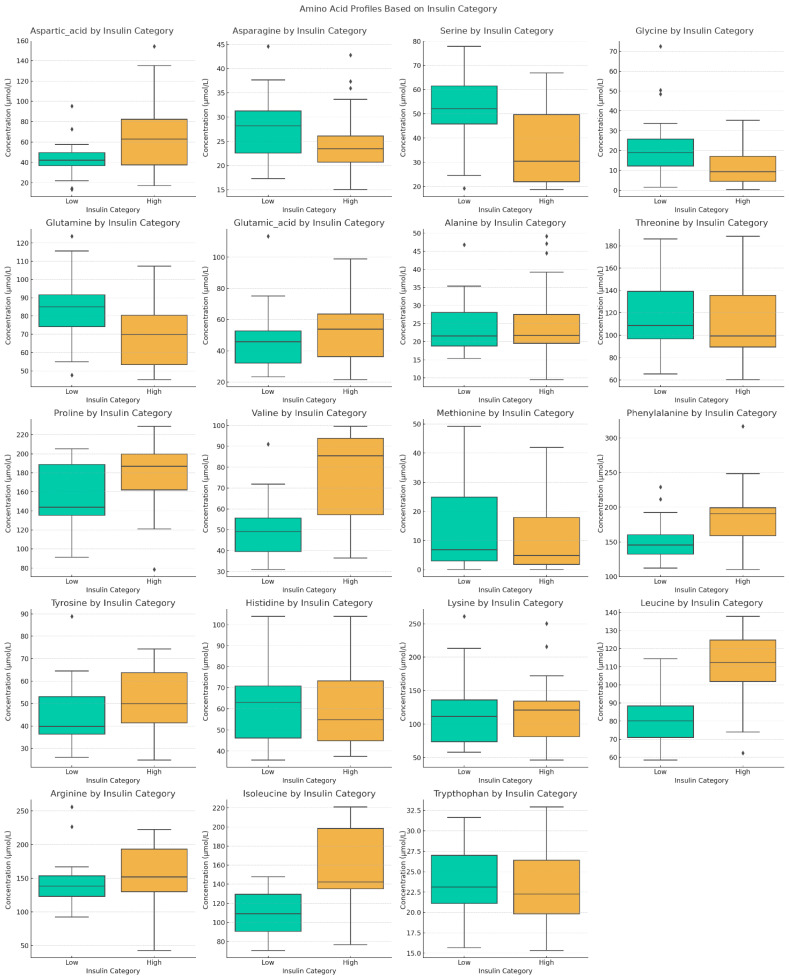
Amino acid profiles among BMI, HOMA-IR, and insulin categories. Results presented as boxplots, with boxes representing the interquartile range, horizontal lines indicating the median, whiskers depicting data variability (concentration of each amino acid is shown in μmol/L).

The between-group testing across three BMI groups had identified a broad amino-acid signature associated with adiposity (Figure 1). Most significantly for serine (*p* = 3.29 × 10^−8^), leucine (*p* = 1.91 × 10^−7^), glutamine (*p* = 1.54 × 10^−6^), valine (*p* = 3.14 × 10^−6^), aspartic acid (*p* = 3.74 × 10^−5^), isoleucine (*p* = 1.87 × 10^−4^), and proline (*p* = 1.74 × 10^−4^), alongside significant effects for phenylalanine (*p* = 1.16 × 10^−3^), tyrosine (*p* = 1.75 × 10^−3^), histidine (*p* = 2.67 × 10^−2^), and glycine (*p* = 3.56 × 10^−3^). No significant BMI-related differences were observed for alanine, threonine, methionine, lysine, or tryptophan (*p* > 0.05). Obese individuals exhibited higher circulating levels of branched-chain amino acids (BCAAs: valine, leucine, isoleucine) as well as aromatic amino acids (tyrosine, phenylalanine) and glutamic acid, whereas glycine and glutamine were reduced relative to normal-weight and overweight participants. Notably, BCAA and tyrosine elevation was already apparent in the overweight group, suggesting that portions of the amino-acid shift precede obesity.

Similar associations were observed between amino acids and insulin-resistance–related endpoints, showing that BCAAs carry the strongest statistical signals for HOMA-IR (valine *p* = 2.75 × 10^−9^; leucine *p* = 9.36 × 10^−9^; isoleucine *p* = 1.68 × 10^−8^) and that serine (*p* = 5.62 × 10^−3^) also differs across HOMA-IR, with phenylalanine reaching significance as well (*p* = 1.76 × 10^−2^). For fasting insulin, significant associations are again observed for valine (*p* = 8.05 × 10^−7^), leucine (*p* = 1.96 × 10^−6^), isoleucine (*p* = 7.07 × 10^−6^), serine (*p* = 5.10 × 10^−4^), glutamine (*p* = 5.24 × 10^−3^), glycine (*p* = 5.61 × 10^−3^), proline (*p* = 1.50 × 10^−2^), phenylalanine (*p* = 3.90 × 10^−3^), and aspartic acid (*p* = 6.66 × 10^−3^), indicating that the BMI-linked pattern extends to insulin-related phenotypes.

### 2.3. Correlations Between Amino Acid Profiles and Metabolic Markers

**Figure 2:**
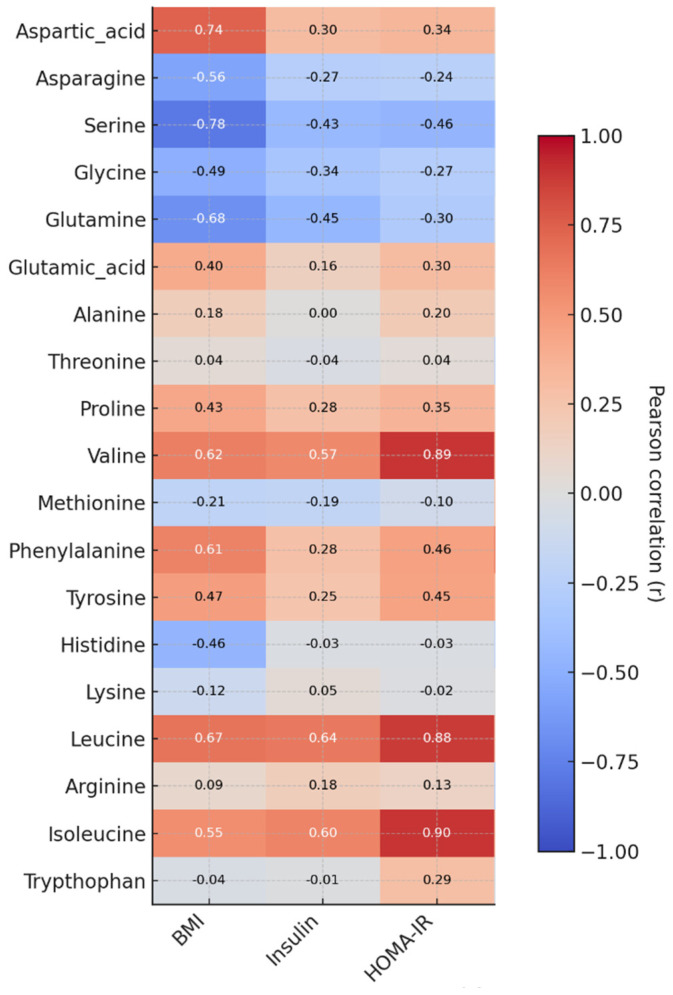
Correlation between amino acid concentrations and chosen variables. Pearson correlation coefficients (r) between 19 circulating amino acids (rows, ordered alphabetically from Alanine to Valine) and chosen metabolic variables (columns: BMI, insulin, HOMA-IR). Positive correlations are shown in red, negative correlations in blue, with the strength of the correlation indicated by color intensity. Exact r values are displayed within each cell.

The correlation analysis revealed distinct and heterogeneous associations between amino acids and metabolic parameters (Figure 2, Table 2). Aspartic acid exhibited strong positive correlations with BMI (r = 0.74). In contrast, serine (r = −0.78) and glutamine (r = −0.68) showed strong negative correlations with BMI. Among the branched-chain amino acids, valine, leucine, and isoleucine showed the highest correlations with HOMA-IR (r = 0.89, r = 0.88, and r = 0.90, respectively) and insulin (r = 0.57, r = 0.64, and r = 0.60, respectively), suggesting a pronounced link with insulin resistance. Several amino acids, such as alanine, threonine, methionine, lysine, and tryptophan, showed generally weak correlations across most metabolic measures, indicating limited or no consistent relationships. Overall, Branched-Chain Amino Acids (BCAAs: Valine, Leucine, Isoleucine) show strong positive correlations with BMI, insulin, and HOMA-IR, indicating their potential role in insulin resistance and obesity. Glutamine and Glycine tend to show negative correlations with insulin resistance markers.

### 2.4. Identifying Amino Acids as Potential Predictors of Insulin Resistance

The Random Forest model and regression analysis were used to determine which amino acids are the strongest predictors of insulin resistance (HOMA-IR). Overall, the predictive performance of Random Forest Model (CV): R^2^ = 0.86 ± 0.06, RMSE = 49.0 ± 8.5 MAE = 39.7 ± 8.0 and the identified top predictors (as permutation importance; mean ΔR^2^ on held-out folds) were as follows: Valine − ΔR^2^ = 0.231 (SD 0.081), Isoleucine − ΔR^2^ = 0.218 (SD 0.077), Leucine − ΔR^2^ = 0.167 (SD 0.052), Tyrosine − ΔR^2^ = 0.015 (SD 0.013), Glutamine/Asparagine − ΔR^2^ ≈ 0.003 each (small; near-zero). The cumulative contribution of BCAAs (Valine + Isoleucine + Leucine) was ΔR^2^ ≈ 0.617 across folds.

The multiple linear regression analysis further confirmed the predictive value of amino acids for insulin resistance. Among the predictors, isoleucine emerged as the strongest determinant of insulin resistance (*p* < 0.001). Both valine and leucine were also significantly and positively associated with HOMA-IR (*p* = 0.035).

### 2.5. Principal Component Analysis Between BMI and Amino Acids Profiles

**Figure 3:**
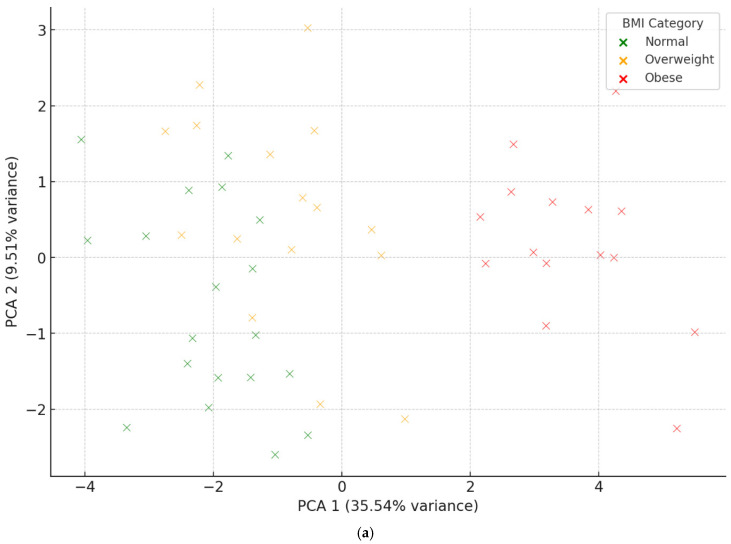

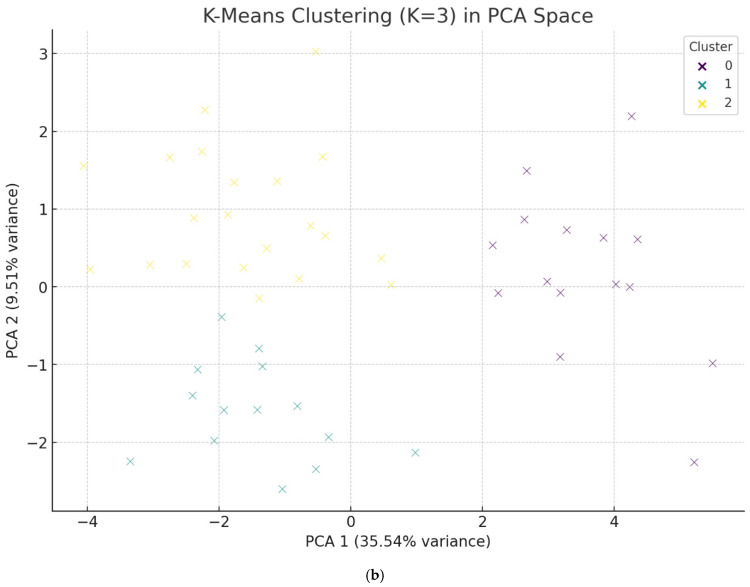
Principal component analysis (PCA) of amino acid profiles (**a**) and K-means clustering of amino acid profiles in PCA space (K = 3) (**b**). Individuals are colored according to BMI category (normal weight, overweight, obese) in the PCA graph and according to clusters in the K-Means graph.

Principal component analysis (PCA) was performed to identify patterns of amino acids linked to BMI categories. The first two components explained 45.05% of the total variance (PC1: 35.54%, PC2: 9.51%). Individuals with obesity and overweight clustered distinctly in the PCA space, while normal-weight participants showed greater dispersion, reflecting more variable amino acid profiles (Figure 3). PC1 was dominated by branched-chain amino acids (BCAAs: isoleucine (0.33), leucine (0.31), valine (0.30) together with serine (0.32) and glutamine (0.31), indicating that obesity-related variance is primarily driven by BCAAs and amino acids linked to energy metabolism. In contrast, PC2 was influenced by threonine (0.47), tryptophan (0.41), asparagine (0.37), histidine (0.32), and alanine (0.31), suggesting this component reflects inter-individual metabolic variation rather than BMI-related shifts.

K-means clustering (K = 3) of the PCA space identified three distinct amino-acid-profiled groups. The K = 3 clustering solution demonstrated improved biological interpretability compared with alternative clustering structures, as it consistently separated participants according to both adiposity and metabolic impairment. The clustering pattern remained stable across repeated algorithm initializations, supporting the robustness of the identified metabolic subgroups. Cluster 0 (Obese Group): comprised exclusively of obese individuals, characterized by the highest BMI, insulin resistance (HOMA-IR), and insulin levels. Cluster 1 (Healthy Group): contained mostly normal-weight individuals, showing the lowest BMI and HOMA-IR. Cluster 2 (Intermediate/Overweight Group): consisted primarily of overweight individuals, with no obese cases. This group demonstrated intermediate BMI, while HOMA-IR and insulin levels were closer to those of the healthy group.

Statistical comparisons confirmed that Cluster 2 differed significantly from Cluster 0 (obese) in BMI (*p* = 5.08 × 10^−8^), and HOMA-IR (*p* = 0.0015), while showing only modest differences from Cluster 1 (healthy). Specifically, insulin resistance in Cluster 2 did not differ significantly from healthy individuals, indicating a “metabolically healthy overweight” subgroup.

**Figure 4:**
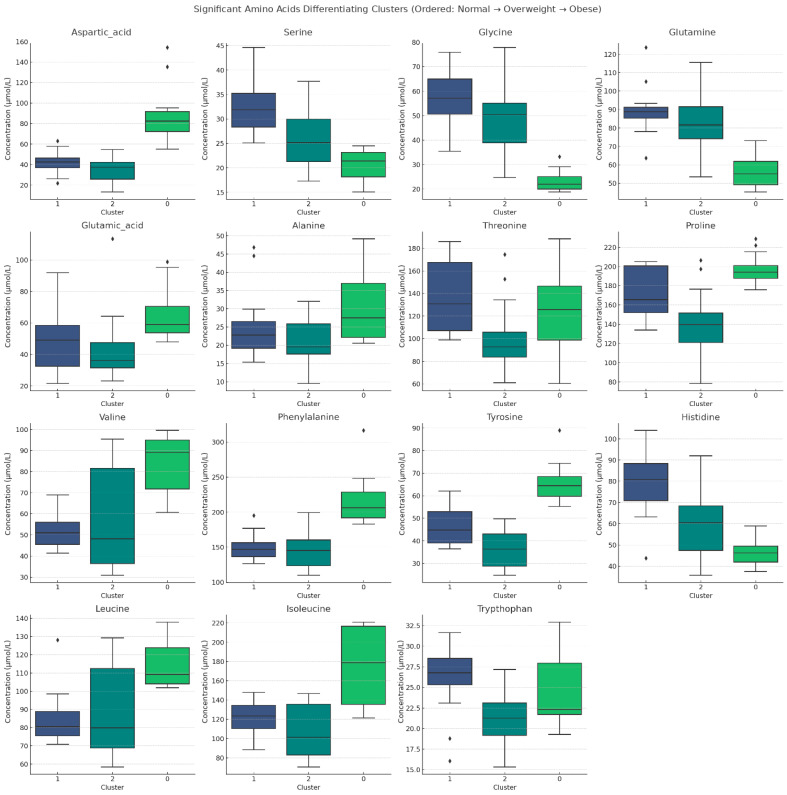
Significant (*p* < 0.05) amino acids differentiating clusters (1—cluster “normal” 2—cluster “intermediate/overweight”, 0—cluster “obese”).

Amino acids significantly contributing to the differentiation between clusters included the following: BCAAs—elevated in both overweight and obese groups, confirming their association with metabolic dysfunction. Glutamine—lower in obese individuals compared to normal-weight and overweight groups. Glutamic acid—markedly increased in obesity, consistent with its role in metabolic stress. Tyrosine and phenylalanine -elevated in obese individuals, with tyrosine already increased in overweight individuals, suggesting an early metabolic shift. Glycine was lowest in obese individuals, consistent with its proposed protective role in glucose and lipid metabolism (Figure 4) [9]. For BCAAs, overweight individuals (Cluster 2) already show elevated levels compared to normal weight. Glutamine is lower in obese individuals compared to both, normal and overweight. Glutamic acid, tyrosine and phenylalanine was significantly increased in obese individuals. Additionally, tyrosine was already elevated in overweight individuals suggesting an early metabolic shift.

## 3. Discussion

Recent advances in metabolomics have revealed that alterations in amino acid metabolism—particularly in branched-chain amino acids (BCAAs)—are closely associated with these metabolic derangements [14]. Profiling the amino acid landscape may thus offer novel insights into the pathophysiology of obesity and enable early identification of individuals at risk for metabolic disorders [13,15].

Branched chain amino acids are indispensable, comprising approximately 35% of essential amino acids in humans [16]. Although plasma levels of other amino acids may also rise in obesity, BCAAs appear to exert unique effects in obesity-induced insulin resistance and are now considered major contributors to the pathogenesis of type 2 diabetes and coronary artery disease [17]. Historical animal studies dating back to the 1960s already established a link between elevated BCAAs and insulin resistance. Human metabolomics echo these results, identifying BCAA-related metabolite signatures correlated with metabolic dysfunction [18]. A landmark metabolomics study suggested that elevated circulating BCAAs could predict insulin resistance and type 2 diabetes up to 20 years before clinical presentation, far earlier than any other marker [19]. More recently, baseline BCAA levels have been associated with the degree of improvement in insulin resistance after weight-loss interventions [17]. These findings emphasize the translational potential of amino acid profiling for early prediction and risk stratification. Despite extensive research, the precise mechanisms linking increased BCAA levels with these conditions remains elusive [20]. Additionally, apart from BCAAs, the relationship between excessive body weight, insulin resistance and the amino acid profile is not clearly established [21]. This study conducted targeted metabolomic profiling of 19 amino acids and assessed their associations with key metabolic parameters—including body mass index (BMI), HOMA-IR (Homeostatic Model Assessment for Insulin Resistance), and fasting insulin. Using analysis of variance (ANOVA), multiple linear regression, and principal component analysis (PCA), we sought to identify specific amino acids predictive of early insulin resistance. Overall, study leveraged multivariate and machine learning approaches to dissect the amino acid metabolic signatures underlying insulin resistance and obesity. Principal component analysis (PCA) revealed that branched-chain amino acids (BCAAs: valine, isoleucine, leucine), along with serine and glutamine, largely accounted for the primary axis of variation (PC1), while aromatic and polar amino acids contributed to secondary variation (PC2). K-means clustering unearthed three metabolically distinct subgroups—normal-weight healthy individuals, metabolically healthy overweight individuals, and obese individuals with marked metabolic dysregulation—reinforcing the concept of heterogeneity within overweight populations. The metabolically “healthy overweight” cluster (Cluster 2) displayed intermediate clinical features and HOMA-IR similar to normal weight individuals, despite BMI elevations. This resonates with concepts of metabolic health resilience in overweight individuals [22]. The ability to classify individuals within the overweight category based on amino acid-driven risk scores could refine prognostic stratification and intervention targeting.

Across multiple analytic platforms—Random Forest modeling, linear regression, and PCA—BCAAs consistently emerged as dominant predictors of insulin resistance (HOMA-IR), with isoleucine showing the strongest association. These findings align with a growing body of literature linking elevated circulating BCAAs with insulin resistance and type 2 diabetes [23,24]. In addition, study by White et al. 2021 showcased that the baseline BCAA concentration is related to the improvement in insulin resistance among participants after a weight loss intervention [11]. Our data further reinforce this paradigm, highlighting the predictive power and pathophysiological relevance of BCAAs in our cohort. Animal studies demonstrated that diets enriched in BCAAs, particularly when combined with high fat intake, accelerate insulin resistance via mTOR activation and inflammatory mechanisms [25]. Mechanistically, BCAAs modulate insulin signaling via mTOR-mediated feedback inhibition of IRS-1, impair glucose uptake in skeletal muscle, and promote ectopic lipid deposition [26]. The structural basis for the unique metabolic behavior of BCAAs lies in the geometry of their branched aliphatic side chains, which confer exquisite substrate specificity on the branched-chain α-keto acid dehydrogenase (BCKDH) complex—the committed, rate-limiting step of BCAA catabolism [11]. Unlike most amino acids, whose initial catabolic step occurs primarily in the liver, BCAAs undergo aminotransferase-mediated transamination predominantly in peripheral tissues (skeletal muscle, adipose tissue, brain) due to low hepatic BCATm expression. In obesity, the BCKDH complex is inactivated via phosphorylation by its dedicated kinase BCKDK, which is upregulated in adipose tissue, creating a tissue-specific catabolic bottleneck and leading to accumulation of branched-chain keto acids and acylcarnitine intermediates (C3, C5) [27]. At the signaling level, the isobutyl side chain of leucine specifically occupies the hydrophobic sensing pocket of Sestrin2, dissociating it from GATOR2 and unleashing mTORC1 kinase activity. Sustained mTORC1 hyperactivation—driven by chronically elevated leucine—promotes serine phosphorylation of IRS-1 and directly impairs insulin signaling [21]. Additionally, a valine-specific catabolic metabolite, 3-hydroxyisobutyrate (3-HIB), acts as a paracrine signal that promotes transendothelial fatty acid transport, linking the valine side chain pathway directly to ectopic lipid deposition and lipotoxic insulin resistance [28].

Amino acids such as arginine and glutamic acid influence endothelial nitric oxide synthesis and vascular tone, thereby linking amino acid dysregulation to early hypertensive changes [7]. Furthermore, aromatic amino acids (phenylalanine, tyrosine) have been associated with subclinical inflammation and beta-cell stress, whereas glycine exerts anti-oxidative and insulin-sensitizing effects [29]. Despite these insights, few studies have interrogated a broad panel of amino acids, comorbidity-free individuals across the full BMI spectrum, limiting the ability to pinpoint early metabolic shifts.

Our regression analysis revealed that BCAAs are significant positive predictors of insulin resistance, with valine, leucine, and isoleucine demonstrating strong statistical associations with HOMA-IR values. Furthermore, PCA illustrates that obesity is associated with a distinct clustering in amino acid space, primarily driven by elevations in BCAAs and glutamic acid.

Beyond BCAAs, aromatic amino acids (phenylalanine, tyrosine) and acidic metabolites like α-hydroxybutyrate and 2-aminoadipic acid (2-AAA) have been implicated in early insulin resistance and predictive of future T2DM [30]. Meanwhile, reductions in glycine and glutamine may reflect compromised metabolic flexibility and antioxidant capacity [31]. Interestingly, in this study, tyrosine demonstrated a significant inverse association with insulin resistance. While aromatic amino acids, particularly phenylalanine and tyrosine, have frequently been linked to metabolic disorders and worse outcomes. In a study of individuals with T2D treated with metformin or placebo, Wang et al. noted a decrease in the plasma levels of carnitine, tyrosine, and valine and an increase in leucine and isoleucine levels with change in insulin levels [19]. Whether these metabolites reflect a response to change in insulin resistance and hyperglycemia or are themselves causative for the change is an area of intense research.

The elevation of phenylalanine and tyrosine alongside BCAAs in our data reflects a convergent structural property: all five are large neutral amino acids (LNAAs) with high hydrophobic character, transported via System L (LAT1/LAT2) carriers that recognize bulky aliphatic or aromatic side chains [32]. Hydrophobicity may therefore constitute a shared predisposition to accumulation when transport is upregulated and sidechain-specific catabolic enzymes are impaired [33]. However, the aromatic ring introduces distinct pathways: phenylalanine hydroxylase (PAH)-mediated conversion of Phe to Tyr requires tetrahydrobiopterin (BH^4^), whose bioavailability is reduced by obesity-associated oxidative stress, while tyrosine’s role as a precursor of catecholamines and thyroid hormones introduces additional regulatory complexity that may underlie its negative regression association with HOMA-IR [34]. The absence of tryptophan elevation, despite sharing the same transport system, further illustrates that aromaticity per se does not determine accumulation: obesity-induced upregulation of indoleamine 2,3-dioxygenase (IDO) channels tryptophan into the kynurenine pathway, preventing its systemic rise [35]. Taken together, hydrophobicity predicts co-elevation at the transport level, while specific sidechain geometry and enzyme context determine the magnitude, directionality, and mechanistic consequences of each amino acid’s association with insulin resistance [36].

Taken together, the study corroborates and extends prior metabolomic findings, offering a multivariate framework to classify individuals based on amino acid profiles and associated metabolic risks [28]. These findings have implications for personalized risk stratification and the potential development of amino acid-targeted interventions in metabolic disease prevention. Importantly, the use of multivariate statistical techniques enables the integration of multiple biomarkers into a cohesive framework for risk stratification, facilitating the identification of at-risk individuals prior to the onset of overt disease. Such biomarker profiling could inform the development of personalized interventions—including dietary modulation, pharmacological targeting of amino acid metabolism, or microbiota-directed therapies—to restore amino acid homeostasis and mitigate the trajectory toward T2DM and cardiometabolic disease. However, a limitation of our study concerns the sex composition of the current cohort. The study population was predominantly female, which precluded adequately powered sex-stratified analyses. Future studies in larger, sex-balanced cohorts should formally account for sex as a biological variable when profiling amino acid signatures of obesity and insulin resistance. Due to the exploratory nature of clustering analyses and the modest cohort size, the identified metabolic subgroups should be interpreted cautiously until replicated in larger independent populations.

## 4. Materials and Methods

### 4.1. Data Collection

The recruitment for the study was conducted between January 2020 and September 2022. The subjects were recruited through the physiotherapy clinic and the Department and Clinic of Cardiology and Internal Medicine of the Institute of Maritime and Tropical Medicine in Gdynia, Poland, as well as via social media. Overall, 100 patients were enrolled in the study, 80 of whom were enrolled at the qualification stage, and 20 patients were excluded during the study. Sixteen patients were excluded due to their inability to participate in all stages for reasons related to infection, ten due to the lack of one or more study endpoint (e.g., serum sample, survey etc.), and four for no reason provided. As a result, 50 subjects completed all stages of the study. Patients with a history of diabetes mellitus, those taking medications that may modify glucose metabolism, those with uncontrolled hypertension, clinically significant arrhythmias, deep and superficial vein thrombosis, acute kidney injury, acute liver failure, malignancies and autoimmune and infectious diseases were excluded from the study. The patients were divided into three groups. Group I included 20 patients with a normal body mass index (BMI 22 ± 1.5 kg/m^2^, age 38 ± 11 years), group II included 20 overweight patients (BMI 28 ± 1.5 kg/m^2^, age 37 ± 12 years), and group III included 10 obese patients (BMI 34 ± 2 kg/m^2^, age 39 ± 11 years). Eighteen women and two men were included in group I, sixteen women and four men were included in group II, and nine women and one man were included in group III. All subjects received oral and written information about the study, and written informed consent forms were obtained prior to study inclusion.

The study was approved by the Bioethics Committee of the Medical University of Gdańsk (consent No. NKBBN/692/2019–2020; date of approval: 30 January 2020), and the study was conducted in accordance with the principles of the Declaration of Helsinki after the amendment in 2013.

### 4.2. Anthropometric and Blood Pressure Measurements

Anthropometric measurements were performed on all patients using the same scale to determine BMI and waist–hip ratio (WHR). To assess bioelectrical impedance, the Tanita SC-240 body composition analyzer (Tanita Corporation, Tokyo, Japan) was used. In addition, blood pressure was checked for all patients. The detailed procedures for anthropometric measurement, experimental design, bioelectrical impedance assessment and blood pressure assessment have been described in detail in our previous publications [37,38].

### 4.3. Sample Collection and Laboratory Analyses

Following qualification, blood samples were collected to assess the concentration of FPG and insulin. Insulin levels were determined using chemiluminescence (Cobas e601; Roche, Switzerland). Insulin resistance was assessed by calculating the homeostasis of the insulin resistance assessment model (HOMA-IR) according to the following formula: HOMA-IR = (FPG × fasting serum insulin)/405, assuming < 2 as normal in adults. The FPG and fasting serum insulin units for HOMA-IR calculation were mg/dL and µIU/mL, respectively.

### 4.4. Amino Acid Concentration Analysis

Blood was extracted with acetonitrile (120 µL) with the addition of an internal standard solution (2-chloroadenosine), and incubated on ice for 30 min. After incubation, the samples were centrifuged for 30 min at 14,000 rpm/4 °C. The collected supernatant in the new Eppendorf was frozen and lyophilized. The obtained pellet was dissolved in 100 µL of water and centrifuged 15 min at 14,000 rpm/4 °C. The concentration of amino acids in the plasma was determined by high performance liquid chromatography-mass spectrometry (LC/MS). Analyzed 19 amino acids and IS (internal standard) were identified and confirmed by ion mass similarity, fragmentation pattern and chromatographic retention time processed with Xcalibur 2.1 program [39].

### 4.5. Statistical Analysis

All statistical analyses were conducted using R programming language (version 4.3). The study included 50 participants with complete anthropometric and biochemical characterization. Amino acid quantification yielded 19 distinct metabolites per individual. Data are presented as mean ± standard deviation (SD) unless otherwise specified. Between-group differences in amino acid concentrations across BMI categories (normal weight, overweight, obese) were evaluated using one-way analysis of variance (ANOVA), followed by pairwise t-tests with Bonferroni correction for multiple comparisons.

Multivariate dimensionality reduction was performed using principal component analysis (PCA) on z-standardized amino acid concentrations. The proportion of variance explained by each principal component was calculated, and loading coefficients were used to identify amino acids contributing most strongly to each component. To determine the optimal number of clusters, several clustering solutions (K = 2–5) were evaluated using the elbow method and average silhouette width. The K = 3 solution was selected because it provided the best balance between cluster separation, biological interpretability, and within-cluster homogeneity. Specifically, the K = 3 configuration distinguished normal-weight individuals, overweight individuals with relatively preserved metabolic characteristics, and obese individuals with pronounced metabolic dysregulation.

Cluster stability was additionally assessed through repeated initialization of the K-means algorithm using multiple random starting centroids (nstart = 25) to minimize sensitivity to local minima and improve reproducibility of cluster assignments.

K-means clustering (K = 3) was applied in the PCA space to classify participants into metabolic subgroups. Cluster assignments were compared against clinical variables (BMI, HOMA-IR, insulin) using ANOVA and independent t-tests.

Associations between amino acids and insulin resistance (HOMA-IR) were further examined using multiple linear regression. The full model included all amino acids as predictors, and variance explained was quantified by the coefficient of determination (R^2^). Regression coefficients and p-values were reported for each amino acid, with significance defined as *p* < 0.05.

Machine learning models were developed to predict both continuous (HOMA-IR) and categorical outcomes (cluster assignment, obesity risk). Random Forest regression was applied to predict HOMA-IR using amino acid profiles, with hyperparameters set to 1000 trees and default feature sampling. Model performance was evaluated using five-fold cross-validation, reporting mean R^2^, root mean squared error (RMSE), and mean absolute error (MAE) with standard deviations. Variable importance was assessed using both mean decrease in impurity (MDI) and permutation importance across cross-validation folds. A Random Forest classifier was additionally trained to discriminate overweight individuals who were metabolically stable from those at risk of transitioning to obesity. Classification accuracy, precision, recall, and F1-scores were computed, and confusion matrices were generated. All tests were two-sided, and statistical significance was set at *p* < 0.05. Prior to machine learning analyses, amino acid concentrations were z-standardized to ensure comparability across variables with different concentration ranges. Random Forest regression and classification analyses were performed using the Random Forest and caret packages in R (randomForest (version 4.7-1.2); caret (Version: 7.0-1). For Random Forest regression predicting HOMA-IR, model hyperparameters included 1000 trees (ntree = 1000) and default feature sampling (mtry = p/3, where p represents the number of predictors). Model performance was evaluated using repeated five-fold cross-validation to reduce overfitting and estimate generalizability. During each cross-validation iteration, the dataset was randomly partitioned into training (80%) and validation (20%) subsets while maintaining balanced representation of BMI categories.

Model performance was quantified using coefficient of determination (R^2^), root mean squared error (RMSE), and mean absolute error (MAE), reported as mean ± standard deviation across validation folds. Variable importance was assessed using both mean decrease in impurity (MDI) and permutation importance. Permutation importance was calculated by randomly shuffling each predictor within held-out validation folds and quantifying the resulting decrease in predictive performance (ΔR^2^). To improve reproducibility, analyses were conducted using a fixed random seed (set.seed(123)).

In addition, Random Forest classification models were developed to distinguish metabolically stable overweight individuals from individuals displaying obesity-associated metabolic profiles. Classification performance was assessed using accuracy, precision, recall, F1-score, and confusion matrices. Given the relatively small cohort size, machine learning analyses were considered exploratory and hypothesis-generating rather than definitive predictive models.

## 5. Conclusions

This study demonstrates that circulating amino acid profiles, particularly branched-chain amino acids (BCAAs: valine, isoleucine, leucine), provide strong discriminatory and predictive value for insulin resistance and obesity. Multivariate analyses (PCA and clustering) revealed that BCAAs dominate the principal axis of metabolic variation and reliably segregate obese from non-obese individuals. Machine learning (Random Forest) and multiple linear regression models consistently identified BCAAs as the most influential predictors of HOMA-IR, with isoleucine emerging as the strongest single determinant.

Beyond BCAAs, tyrosine displayed a significant negative association with insulin resistance, suggesting a potential counter-regulatory or protective role that merits mechanistic validation. Clustering analyses further identified a distinct subgroup of overweight individuals with preserved metabolic health, underscoring the heterogeneity of the overweight state and the value of amino acid profiling in stratifying risk.

Collectively, these findings establish amino acid metabolism—most prominently BCAAs—as a potential early biomarkers of insulin resistance and obesity risk. The reproducibility of these associations across complementary analytic frameworks provides robust evidence that amino acid signatures may serve both as biomarkers for early metabolic deterioration and as potential therapeutic targets. Future longitudinal studies are required to confirm their causal role and to evaluate whether interventions modulating amino acid balance can alter trajectories toward metabolic disease.

## Figures and Tables

**Table 1:** Clinical characteristics of patients with normal body mass index (group I), overweight patients (group II), obese patients (group III) [BMI—Body mass Index, SBP—systolic blood pressure, DBP—diastolic blood pressure, WHR—waist-hips ratio, VAT—visceral adipose tissue].

Measured Parameter	Group I	Group II	Group III	Group Comparison	*p* Value
Age [years]	39 ± 11	39 ± 13	39 ± 11	I vs. II I vs. III II vs. III	ns ns ns
BMI [kg/m^2^]	22 ± 2	28 ± 2	34 ± 2	I vs. II I vs. III II vs. III	0.016 0.014 0.0001
SBP [mmHg]	121 ± 2	126 ± 3	140 ± 4	I vs. II I vs. III II vs. III	ns 0.000000007 0.0006
DBP [mmHg]	74 ± 6	81 ± 5	87 ± 7	I vs. II I vs. III II vs. III	ns ns ns
WHR	0.8 ± 0.06	1 ± 0.04	1.2 ± 0.08	I vs. II I vs. III II vs. III	0.00001 0.0000003 0.0000002
VAT [LVL]	4 ± 2	7 ± 3	9 ± 2	I vs. II I vs. III II vs. III	0.0015 0.001 0.008

Data presented as mean values and their standard deviations; *p*-value—significant difference (*p* < 0.05). ns—not significant.

**Table 2:** The relationships between BMI, HOMA-IR, insulin, and the concentration of 19 amino acids. [* marks significant difference *p* < 0.05 after one-way analysis of variance (ANOVA), followed by pairwise *t*-tests with Bonferroni correction for multiple comparisons].

Metabolic Marker	Amino Acid	*p* Value
BMI [kg/m^2^]	Aspartic_acid	3.74 × 10^−5^ *
	Asparagine	0.002964006 *
	Serine	3.29 × 10^−8^ *
	Glycine	0.003564444 *
	Glutamine	1.54 × 10^−6^ *
	Glutamic_acid	0.017576275 *
	Alanine	0.138940922 *
	Threonine	**0.909406516**
	Proline	0.000173977 *
	Valine	3.14 × 10^−6^ *
	Methionine	0.138547401
	Phenylalanine	0.001155241 *
	Tyrosine	0.001744651 *
	Histidine	0.02672964 *
	Lysine	0.979750248
	Leucine	1.91 × 10^−7^ *
	Arginine	0.137442094
	Isoleucine	0.000187167 *
	Trypthophan	0.963655302
Insulin [µIU/mL]	Aspartic_acid	0.006658748 *
	Asparagine	0.090167328
	Serine	0.000510011 *
	Glycine	0.005612748 *
	Glutamine	0.005239725 *
	Glutamic_acid	0.142779233
	Alanine	0.709140709
	Threonine	0.466404944
	Proline	0.015023665 *
	Valine	8.05 × 10^−7^ *
	Methionine	0.540073917
	Phenylalanine	0.003903808 *
	Tyrosine	0.141528551
	Histidine	0.85730746
	Lysine	0.906314399
	Leucine	1.96 × 10^−6^ *
	Arginine	0.274680447
	Isoleucine	7.07 × 10^−6^ *
	Trypthophan	0.700227166
HOMA-IR	Aspartic_acid	0.095237106
	Asparagine	0.559963076
	Serine	0.005622753 *
	Glycine	0.0056134804 *
	Glutamine	0.105559341
	Glutamic_acid	0.082267853
	Alanine	0.547601353
	Threonine	0.859751482
	Proline	0.052088037
	Valine	2.75 × 10^−9^ *
	Methionine	0.419803385
	Phenylalanine	0.017613641 *
	Tyrosine	0.07549855
	Histidine	0.502061695
	Lysine	0.766467591
	Leucine	9.36 × 10^−9^ *
	Arginine	0.206463837
	Isoleucine	1.68 × 10^−8^ *
	Trypthophan	0.324690354

## Data Availability

The original contributions presented in this study are included in the article. Further inquiries can be directed to the corresponding author.
